# The learning preferences of millennial emergency medicine residents in Qatar

**DOI:** 10.5116/ijme.5d1b.ae92

**Published:** 2019-07-27

**Authors:** Khalid Bashir, Kaleelullah Saleem Farook, Stephen H. Thomas

**Affiliations:** 1 Department of Emergency medicine, Hamad General Hospital, Doha, Qatar

**Keywords:** Learning preferences, millennial, emergency medicine, residents, Qatar

## To the Editor

The purpose of this letter is to describe our experiences of the learning preferences of millennial residents in an emergency medicine (EM) program. Millennials, also known as Generation Y, are those  born between 1981 and 1996, and aged 23-33 in 2019. From a pedagogical perspective, millennials have a different mindset compared to the previous generations (for example, generation X, born between 1960 and 1980). They are ‘tech-savvy’ learners who tend to use social networking services or social media. In addition, they tend to team-based learning and ‘ a flat hierarchy’.^1^  Because of this, millennials may use social media as a learning tool in medicine, rather than the conventional methods of teaching.^2^ It has been shown that different generations  (e.g., Generation X and Y)  have different learning style preferences.  Given the learning preferences of millennials,  many medical schools in Europe and the United States have developed distance and online courses, problem-based learning and flipped classrooms for their undergraduate and post-graduation medical education.^3^^,^^4^ However, questions have been raised about the learning preferences of millennials in non-western countries, especially in Arab students who have different scoiculture perspectives.  Therefore, we felt it important to investigate the learning preferences of our millennials learners. To achieve this,  we conducted a quasi-experimental study of EM residents to examine their learning preferences of the diagnosis and management of benign paroxysmal positional vertigo (BPPV), which is most common in emergency departments.^5^ Thirty-eight EM residents (13 women and 25 men)  were randomly assigned to two groups. The age range, experiences, and prior knowledge of BPPV were more or less the same among all residents. The first group was exposed to a traditional didactic lecture followed by supervised practice of the physical treatment maneuvers. The second group was exposed to a blended learning approach wherein the residents watched an online lecture and a video of the maneuvers and then practiced with each other without any support from clinical supervisors. Twenty-five out of 38 EM residents preferred traditional didactic lecture over blended learning,  and it was statistically significant. A possible explanation for this might be attributed to the fact that  EM residents have graduated from medical schools in the Middle East and Asia where traditional methodologies are strongly associated with the teaching and practice of medicine.   The old methods of teaching in undergraduate medical education may result in medical residents prefering the same methods for learning and practice rather than contemporary methods in postgraduate medical education.^6^

Although the results of this study should be interpreted cautiously, Middle Eastern medical educators should be aware of special importance given to the application of modern teaching techniques in medical education, especially for millennials who grew up with computers. In addition, academic staff may equip themselves with new approaches to teaching and learning in order to involve students with self-directed learning using online and distance courses. Although the current study is based on a small sample of EM residents,  our findings are not consistent with those of other studies in Europe and the US^7^^,^^8^  this raises a possible concern for medical educationists in the Middle East to improve teaching and learning in postgraduate medical education. Although medical education is consistently evolving, especially teaching methods and strategies, we may not assume that millennials will automatically involve online courses and self-directed learning in Middle East countries. Therefore, we recommend further medical education research in Middle East training programs to examine the changing attitudes of millennials in graduate medical education.

### Conflict of Interest

The author declares that there are no conflicts of interest.
